# Matchmaking in Bioinformatics

**DOI:** 10.12688/f1000research.13705.1

**Published:** 2018-02-09

**Authors:** Ewy Mathé, Ben Busby, Helen Piontkivska, Team of Developers

**Affiliations:** 1 Department of Biomedical Informatics, College of Medicine, The Ohio State University, Columbus, OH, 43210, USA; 2 National Center for Biotechnology Information (NCBI), National Library of Medicine, National Institutes of Health, Bethesda, MD, 20894, USA; 3 Department of Biological Sciences and School of Biomedical Sciences, Kent State University, Kent, OH, 44242, USA

**Keywords:** computational biology, bioinformatics, biology, speed dating, collaboration, matchmaking

## Abstract

Ever return from a meeting feeling elated by all those exciting talks, yet unsure how all those presented glamorous and/or exciting tools can be useful in your research?  Or do you have a great piece of software you want to share, yet only a handful of people visited your poster? We have all been there, and that is why we organized the Matchmaking for Computational and Experimental Biologists Session at the latest ISCB/GLBIO’2017 meeting in Chicago (May 15-17, 2017). The session exemplifies a novel approach, mimicking “matchmaking”, to encouraging communication, making connections and fostering collaborations between computational and non-computational biologists. More specifically, the session facilitates face-to-face communication between researchers with similar or differing research interests, which we feel are critical for promoting productive discussions and collaborations.  To accomplish this, three short scheduled talks were delivered, focusing on RNA-seq, integration of clinical and genomic data, and chromatin accessibility analyses.  Next, small-table developer-led discussions, modeled after speed-dating, enabled each developer (including the speakers) to introduce a specific tool and to engage potential users or other developers around the table.  Notably, we asked the audience whether any other tool developers would want to showcase their tool and we thus added four developers as moderators of these small-table discussions.  Given the positive feedback from the tool developers, we feel that this type of session is an effective approach for promoting valuable scientific discussion, and is particularly helpful in the context of conferences where the number of participants and activities could hamper such interactions.

## Introduction

Informal, face-to-face communication between participants is a vital piece of a scientific conference, just as important, if not more important, as formal activities such as keynote addresses and formal talk sessions ( Saunders *et al*., 2009). However, as the number of attendees grows, coupled with multiple research plenary sessions that often run concurrently (a regular feature of conferences in bioinformatics and other fields), the time available for individual contact with conference participants drops dramatically. Further, for new attendees, it can be difficult to navigate abstracts, posters, and talks to figure out the key people to engage with. While social media interactions via Twitter and other similar social media platforms ( Biospace, 2009; Saunders *et al*., 2009; Tachibana, 2014), or dedicated online communities ( Budd *et al*., 2015) have their own role in facilitating conversations, face-to-face conversations remain invaluable ( Budd *et al*., 2015; Fuller *et al*., 2013).

Even for those of us who conduct most of our interactions online, face-to-face interactions can solidify relationships, spur novel ideas and research directions, further promote collaborations, and speed up project implementations. Moreover, it is critical for tool developers to carefully assess the utility (e.g., is their tool addressing an unmet need?) and usability (e.g., how streamlined and simple to use is the tool?) of their software. In the open source community especially, these aspects often tend to be overlooked or there are not enough resources to implement them ( Al-Ageel *et al*., 2015). To assess utility and usability, developers need to establish a network of potential users, and need to get direct input from those users, including whether the software is sufficiently user-friendly to enable the user to focus on hypothesis- generation and testing in lieu of tool tweaking ( Kumar & Dudley, 2007). These interactions can be key in addressing specific needs and/or offering a vision and/or a wish-list for further development (e.g., addition of new features). 

For users, another source for finding tools of interest is via formal publication (peer-reviewed). However, this avenue is relatively slow, and is occasionally inefficient and/or insufficient in reaching a broader audience. Pre-peer-reviewed venues, e.g. bioRxiv, Figshare ( Huang & Lapp, 2013), Zenodo, are trying to address this gap. Nonetheless, often the user’s needs are not well articulated (or even formalized), and that’s where face-to-face discussions can be much more helpful. 

## Developing novel tools that are usable to the wider community

While many tools are being developed, a relatively fewer number are routinely used by the larger biological and medical community. In fact, the average lifespan of an open-source Bioinformatics software is often relatively short, frequently limited by the transient nature of work contracts of developers, many of whom are post-docs or graduate students ( Ahmed *et al*., 2014). Through literature mining, a recent study reported that many database and software resources are mentioned only in the Bioinformatics literature, while only a fraction of the tools are mentioned in the biological and medical literature ( Duck *et al*., 2016). Specifically, only 5% of the resources account for 47% of total usage and over 70% of the resources are only mentioned once in the literature ( Duck *et al*., 2016). This striking bias suggests that while the Bioinformatics community promotes development of novel software, the biological and medical communities only access a fraction of what is available. It is quite reasonable to think that these latter communities only access software that are intuitive and usable, and that perhaps usability could trump accuracy of analyses performed ( Huang & Lapp, 2013; Pavelin *et al*., 2012). 

Of note, two broad approaches could be undertaken when developing Bioinformatics software. First, developers can develop a tool that solves a known issue in the field (e.g. RNA-seq analysis, omics integration), and then can seek users and data to test their approach and software. With this approach, it may be difficult for their tool to have visibility outside the Bioinformatics community, since 1) it is less likely that non-computational users are aware of your tool, and/or 2) your tool may not be user-friendly to non-computational users, and/or 3) your tool may not be readily adaptable to answer specific biological questions, or to accommodate a specific dataset format. With the surge in volume and variety of data types in high-dimensional biological data, adaptability is becoming more and more of a challenge. For example, a novel tool that integrates high-throughput omics data that is collected in the same samples may not be readily adaptable to data that is collected in different samples. Second, developers can develop Bioinformatics solutions that try to answer a specific biological or biomedical question, and can then broaden the utility of the tool by developing an associated software. Because the emphasis is on the biology, the resources and time available to generalize the software to other datasets are oftentimes lacking. This often results in a gap between a goal of developing a user-friendly software and ‘on the ground’ availability of low-level computational infrastructure (which is frequently scripting based) ( Kumar & Dudley, 2007). We believe that this gap could be narrowed by further communication between biologists, computational biologists, clinicians, and users.

Importantly, it is worth noting that developers of widely adopted tools have often formally assessed utility and usability, enabling them to broadly disseminate their software. Guidelines for adopting a user-centered design when developing software have been formally assessed ( Ahmed *et al*., 2014; Pavelin *et al*., 2012), and if applied, could yield highly usable software and could facilitate novel scientific discoveries. These formal assessments typically require face-to-face meetings between developers and users, and require developers to understand what problems need to be addressed, and how users will interact with the software. While taking these aspects into consideration prior to developing software can be lengthy, the resulting software will surely be useful and used by a wider community. Creating useful software can also provide a lot of job satisfaction to developers.

## Reproducibility and software in biomedical research

Creating sustainable computational solutions can have a strong, positive impact on reproducibility of analysis results. With the recent rising concerns in reproducibility of scientific research ( Clark, 2017; Editorial, 2016), it is critically important to ensure that the analysis of large biological datasets is reproducible. More often than not, it is difficult to reproduce graphs and results in publications, and this is largely due to incomplete methods (e.g. parameters missing for statistical methods used, manual curation of results, etc.), and the use of in-house scripts or software. Methods for increasing computational reproducibility include reporting code and documentation used, and automating research analyses ( Piccolo & Frampton, 2016). Computational frameworks, including but not limited to Taverna ( Hull *et al*., 2006; Wolstencroft *et al*., 2013), Galaxy ( Goecks *et al*., 2010) and R markdown ( Baumer & Udwin, 2015; Baumer *et al*., 2014), facilitate reproducibility and oftentimes create reports that record all parameters used during the analysis. In addition to usability, developers can thus take into account the importance of reproducibility and in talking with users, better understand which parameters and analysis information needs to be reported.

## ISCB/GLBIO’2017 conference

Hosted by the University of Illinois at Chicago, International Society for Computational Biology affiliate meeting, Great Lakes Bioinformatics Conference (ISCB/GLBIO’2017), has attracted a record 347 registered participants, including ~60% graduate students and post-docs with a broad range of computational and experimental expertise. First convened in 2006 as the Ohio Collaborative Conferences on Bioinformatics (OCCBIO), since 2010 joining forces with ISCB, over the years GLBIO has established itself as an ideal conference for showcasing the latest developments in analysis approaches and tools that span many different fields, and is a venue that attracts both computational and bench scientists. As we are all aware though, communication between computational and bench scientists can be challenging, particularly during the initial introduction stages when the overlap in mutual interests is not clear, and the matchmaking session that we ran is a first attempt at promoting such communication. 

As Dr. Funmi Olopade (University of Chicago) mentioned in her keynote speech, clinicians, basic researchers, and computational biologists must better communicate to advance research. This sentiment is generally shared in the biological sciences, yet each field has its own language and culture. Encouraging communication across different fields via a common theme (e.g. RNA-seq analysis, chromatin accessibility analysis, etc.) is precisely what our matchmaking session aimed to accomplish. 

## Matchmaking for Computational and Experimental Biologists Session

The Matchmaking Session (Matchmaking@GLBIO session, #GenoMatch, #CompMatchBio) attracted over 40 participants, including 9 tool developers. The session, held at 8 am on the first day of the conference, kicked off with three short introductory talks, followed by multiple rounds of 4–5 minutes long small-table discussions led by individual tool developers, and then open discussion. Short (10 minutes each) introductory talks by Drs. Ben Busby (NCBI), James Chen (OSU) and Ewy Mathé (OSU) covered available NCBI tools for RNA-seq analyses, approaches in integration of clinical and genomic data, and chromatin accessibility analyses, respectively. The purpose of these talks was to introduce broad topics that pose current, relevant topics and challenges in computational biology, and to present developers that are working on tools to address these challenges.

Next, small-table developer-led discussions were modeled after speed-dating. In each round, participants joined a table, listened to the developer’s pitch, asked questions, discovered common interests, exchanged contact information, and then moved on to the next table. Because these small-table discussions were timed (4–5 minutes each), each participant had an opportunity to visit all the tables. At the end of “speed-dating” small-table discussions, participants still had 30–45 minutes available for further discussion. At this point, most users had identified developers that were presenting tools useful to them, and thus had the opportunity to discuss their own data needs in more detail. 

## Tools and representatives of tool developing teams (developers)

When planning the session, three main themes for tools were considered: analysis of RNA-seq, chromatin accessibility, and omics/multi-dimensional integration. A total 5 representatives of tool developer teams (Ben Busby, James Chen, Ewy Mathé, Arunima Srivastava, and Rick Farouni) were pre-registered for the session. However, at the start of the session, we asked whether other developers were interested in sharing their tool and, thus, were able to include 4 more developers. This near doubling of presenter-participants with a last minute change shows the level of interest that already exists in the community for sharing their tools. Table 1 lists all tools that were presented, with relevant reference information.

**Table 1.** Tools highlighted by developers during the matchmaking session. Each developer had a chance to showcase their tool and to further discuss its usage with potential collaborators during the “speed-dating” small-table discussions.

Tool name	Presenters	Publication/Website
***Clust***: Optimized consensus clustering of one or more heterogeneous gene expression datasets (e.g. Microarrays and RNASeq)	Basel Abu-Jamous and Steven Kelly	https://github.com/BaselAbujamous/clust
***ProcessDriver***: Tools that computes copy-number based cancer drivers and associated dysregulated biological processes ***GSEPD***: An R package to compute differentially expressed genes, enriched GO terms and projection- based clustering of samples	Serdar Bozdag	B. Baur and S. Bozdag. *ProcessDriver: A computational pipeline to identify* *copy number drivers and associated disrupted biological processes in* *cancer.* Genomics, 2017, 109(3–4): 233–240. https://github.com/brittanybaur/ProcessDriver
RNA-seq resources at NCBI	Ben Busby	https://www.ncbi.nlm.nih.gov/guide/dna-rna/
***MatchTX***: An automated learning system for patient cohort matching using high-dimensional genomic data	James Chen	www.match-tx.com
***Kover***: A machine learning tool to learn interpretable models of phenotypes from k-mer data	Alexandre Drouin	https://github.com/aldro61/kover Drouin, A., Giguère, S., Déraspe, M., Marchand, M., Tyers, M., Loo, V. G., ... & Corbeil, J. (2016). *Predictive computational phenotyping and biomarker* *discovery using reference-free genome comparisons*. *BMC genomics*, *17*(1), 754.
***ALTRE***: workflow for defining ALTered Regulatory Elements using chromatin accessibility data	Rick Farouni	https://github.com/mathelab/altre Baskin E., Farouni R. , Mathé E.A. *ALTRE: workflow for defining ALTered* *Regulatory Elements using chromatin accessibility data*. Bioinformatics 2017; 33 (5): 740–742.
***IntLIM***: Integration of metabolomics and gene expression data	Ewy Mathé	https://github.com/mathelab/intlim
***SeqclusterViz***: Small RNASeq visualization	Lorena Pantano	https://github.com/lpantano/seqclusterViz https://f1000research.com/posters/6-673
***OSUMO***: Multi-Omic data utilization and patient stratification	Arunima Srivastava	https://github.com/osumo/

## Feedback from presenters

As a follow-up to the session, developers were asked about their experiences afterwards, whether they had the sufficient opportunity to discuss their tools with potential users, and whether the subsequent interactions have occurred during the remainder of the conference. The majority of developers have found the session to be quite useful, in part due to the opportunity to network with many potential users, during the session or afterwards. Having time constraints for the matchmaking rounds have also allowed the session participants to quickly determine whether or not they were interested in learning about a specific tool in depth, and if the latter, move on to another tool.

Of note, the 5-minutes rounds were sufficiently long to accommodate exchange of contact information for subsequent follow-up, which occurred later during the conference functions and/or after the conference was over. The primary aim of the session was to provide face-to-face interactions between users and developers, and to provide ample opportunities for contact information exchange. Per feedback we received afterwards, this aim appeared to be successfully accomplished. 

## Future matchmaking sessions

We plan to build up and expand on our successful experiment during GLBIO’2017, to offer similar matchmaking sessions at other ISCB venues, such as ISMB in Chicago in 2018, and GLBIO in 2019 Madison, WI. We have already run an informal session at the ISCB DC-RSG summer workshop in College Park, Maryland (July 12, 2017) with lightweight planning, enormous popularity, and a very positive response.

In the future, to broaden participation and improve participants’ experience, presenters/developers will be given the opportunity to prepare and present 1-2 slides about their tools at the beginning, similar to ‘flash talks’. This format will help developers to find other developers interested in solving similar problems. In our first matchmaking session, developers had little time to interact with each other during the session. In the future this flash talks-format could replace the broad, introductory topic-focused talks given at the beginning of the matchmaking session. Notably, though, these flash talks will not replace the small-table matchmaking portion of the session, which we believe is critical to foster communication between users and developers.

Lastly, it is important to note that this session was scheduled at 8 am at the start of the conference. While we had anticipated lower participation due to this scheduling (assuming that a number of participants would chose to come in later on the first day to avoid traveling the Sunday prior to the start of the conference), the timing of the session turned out to be advantageous. Indeed, having a discussion-promoting, interactive session as a start of the conference is a great way to engage participants and “break the ice” for subsequent interactions during the conference. Further, it provides ample time for attendees to find each other later during the conference and formalize potential collaborations.

## Conclusions

The short-talk/“speed-dating” format provided a platform in which participants could learn about as many tools as possible in a short period of time, while making valuable connections across fields. Given the fast moving pace of Bioinformatics and the rapid advances across clinical/experimental biology fields, it is critical to keep the communication lines open between the communities. Our matchmaking session opened these communication lines by facilitating informal face-to-face interactions.

## Data availability

*All data underlying the results are available as part of the article and no additional source data are required.*
