# Supplementary material for: Matchmaking in Bioinformatics
Source: F1000Res. 2018 Feb 9;7:ISCB Comm J-171. [Version 1] doi: 10.12688/f1000research.13705.1 (PMC5871941; doi:10.12688/f1000research.13705.1)
Supplement: Peer review history

# Peer review: Referee response for version 1

**DOI:** 10.5256/f1000research.14887.r30752

**Published:** 2018-03-27

**Authors:** Guenter Tusch

**Affiliations:** 1 Grand Valley State University, Allendale, MI, USA

The authors discuss a unique experimental session that they initiated at the ISCB/GLBIO’2017 meeting in Chicago (May 15-17, 2017) in form of an opinion article. Based on the model of speed dating they teamed up interested parties with developers of bioinformatics software in order to connect those developers with potential users. The paper consists of roughly four parts. It starts with a brief introduction including a plea for the importance of face-to-face interactions at conferences and a description of options researchers have today to find the appropriate computer software to support their research projects. The next part describes the software development process for bioinformatics software as seen by the authors. They claim that there are basically two approaches that I would call developer-centric and research-centric. The first one seems to assume that developers develop a more general tool, but have difficulties to connect to potential users, while the other one apparently results in a program that suffers from a lack of general usability due to a too narrow focus on a specific biological problem. I’m not quite sure if this a based on the NCBI experience of one of the co-authors and how tools like Bioconductor would fit in here. There is certainly a problem for small scale software projects like those developed for one particular research project. That could be clarified with specific examples possibly from participants in the matchmaking session.

 The next part of the paper describes and discusses the session at the conference, emphasizing the focus on face-to-face communication, setup, implementation, feedback of presenters, and future plans. I thought of this as the essential part of the paper. Finally, as a third part the authors included a table with the description of the presented software and contact information.

 I believe that this experimental session is a very interesting and important approach, and the authors make very valuable points about the setup and implementation of the session and the outcomes especially for younger researcher. From the success the authors had I hope there will be more sessions like that at future conferences. Of course, the conclusions need to be preliminary based on only one session, however the authors make strong points that many results can be generalized. While the introductory and the second part feel like a unit and are the only ones referred to in the conclusion, I feel like the second part and the table are not really integrated enough. They deal with important aspects of the topic, if the topic is not a mere description of the whereabouts of the session and the conclusions drawn by the authors. If the purpose is purely informative, it could be largely reduced, but if it is part of the argument – as I assume, see also my comments above -, it should be included in the discussion and conclusion, and that would strengthen the message. I also agree with the comments of the other reviewer.

 In conclusion, the authors discuss a very interesting and promising approach to improve communication and personal connections especially for younger researcher in the bioinformatics community.

I have read this submission. I believe that I have an appropriate level of expertise to confirm that it is of an acceptable scientific standard.

# Peer review: Referee response for version 1

**DOI:** 10.5256/f1000research.14887.r30750

**Published:** 2018-02-26

**Authors:** Robert M. Blumenthal

**Affiliations:** 1 Department of Medical Microbiology and Immunology, Program in Bioinformatics, University of Toledo College of Medicine and Life Sciences, Toledo, OH, USA

This manuscript summarizes experience and justification for a rapid developer-user meeting format, which was first implemented at the 2017 GLBIO-ISCB meeting. It is a useful summary and may stimulate others to try similar approaches. My comments are entirely on ways to clarify the writing, because the content is fine as is. 

- P3 Para2: The heavy use of “e.g.” is distracting and unnecessary – suggest just leaving it out.
- P3 Para4: Top line, “fewer” should be “smaller”; 3 ^rd^ line delete “an”; 4 ^th^ line delete “often” (since you use the word “average”). Next column (same para), add a comma after “total usage”; near bottom of para replace “are” with “is” before “intuitive”.
- P3 Para5: 3 ^rd^ line delete “e.g.”; 7 ^th^ line, replace “since” with “for one or more of the following reasons:” and delete both occurrences of “and/or”; 12 lines from bottom replace “Second” with “In the second broad approach”; and 3 lines below that remove “an”.
- P3 Para7: replace “analysis” with “analytic”.
- P3 and throughout: Is it F1000Research style to capitalize “Bioinformatics” with every use?
- P4 Para2: top line add “the” before “International”; 3 ^rd^ line remove “has”; 7 ^th^ line add “and” before “since”.
- P4 Para3: remove “e.g.”
- P4 Para7: remove “have” before “also allowed the session”.
- P5 Para2: unclear what is meant by “lightweight” – please clarify.

I have read this submission. I believe that I have an appropriate level of expertise to confirm that it is of an acceptable scientific standard.
